# Insulin Resistance in Association with Thyroid Function, Psychoemotional State, and Cardiovascular Risk Factors

**DOI:** 10.3390/ijerph18073388

**Published:** 2021-03-25

**Authors:** Nijole Kazukauskiene, Aurelija Podlipskyte, Giedrius Varoneckas, Narseta Mickuviene

**Affiliations:** Laboratory of Behavioral Medicine, Neuroscience Institute, Lithuanian University of Health Sciences, LT-00135 Palanga, Lithuania; aurelija.podlipskyte@lsmuni.lt (A.P.); giedrius.varoneckas@lsmuni.lt (G.V.); narseta.mickuviene@lsmuni.lt (N.M.)

**Keywords:** insulin resistance, coronary artery disease, thyroid function, psychoemotional

## Abstract

Background: Individuals with insulin resistance (IR) have a high risk of diabetes or metabolic syndrome, and they are more likely to have depression. Furthermore, IR by itself is a major cardiovascular risk factor in healthy persons. Thus, we aimed to investigate IR in association with thyroid function, psychoemotional state, and cardiovascular risk factors among 45–84-year-old citizens of Palanga. Methods: A randomized epidemiological study was performed with 850 subjects. All participants were evaluated for sociodemographic, clinical, and cardiovascular risk factors and biochemical analysis. IR was evaluated by the homeostasis model assessment of IR (HOMA-IR). Results: All study participants were stratified into groups without IR (HOMA-IR ≤ 2.7) and with IR (HOMA-IR > 2.7). The analysis of parameters between the two study groups showed some statistically significant relationships between IR and cardiovascular risk factors. The predictable accuracy was presented using receiver performance characteristic curves for HOMA-IR scores in women and men separately. If the HOMA-IR score is higher than 3.45, individuals are significantly more likely to have type 2 diabetes mellitus (T2DM). Conclusions: An increase of fasting glucose and more frequent incidence of metabolic syndrome, diabetes, and cardiovascular diseases in subjects with IR are associated with the prevalence of cardiovascular risk factors. There was no significant association between thyroid function and HOMA-IR. HOMA-IR cut-offs could predict the presence of T2DM.

## 1. Introduction

Insulin resistance (IR) occurs when insulin no longer effectively stimulates glucose uptake in metabolic tissues. The inability of metabolic tissues to take up glucose results in hyperglycemia and hyperinsulinemia, both hallmark symptoms of IR. Most individuals with IR go undiagnosed, and the condition can continue for 10–12 years, which can be especially damaging as IR is an independent risk factor for obesity, cardiovascular disease, arterial hypertension (AH), and type 2 diabetes mellitus (T2DM). This time span is an important intervention window where it is possible to prevent and reverse progression towards metabolic disease and T2DM [1,2,3].

In daily clinical practice, the determination of IR indices is based on measurements of fasting glucose and insulin concentrations. Homoeostatic model assessment of IR (HOMA-IR) is the most commonly used indirect indices for defining this condition in the “steady state” [4,5]. Thyroid hormones play a key role in the regulation of energy homoeostasis and thermogenesis and can thereby influence body composition [6,7,8]. Hypothyroidism is associated with weight gain, while hyperthyroidism is associated with higher metabolic rates and weight loss [9]. Changes in thyroid function can lead to the development of metabolic complications, even when thyroid hormone levels are within the normal range [10]. IR, measured using the HOMA-IR, was positively associated with serum free triiodothyronine (FT3) levels in previous studies [7,11]. Higher IR was positively associated with serum FT3 levels and inversely associated with serum thyroid stimulating hormone (TSH) levels in euthyroid subjects with normal thyroid ultrasound findings [12]. However, there were no significant associations between HOMA-IR and serum free tetraiodothyronine (FT4) or TSH levels in other studies [7].

The etiology of metabolic disorders is complex and multifactorial. The key causes are obesity, physical inactivity, sedentary lifestyle, and aging population. Recently, the role of psychological disorders on metabolic parameters is being increasingly recognized [13]. So far, a correlation between IR and depression has been documented in several studies. A study by Adriaanse et al. (2006) [14] consisting of 541 participants (55–75 years old) showed that IR was weakly associated with depressive symptoms. According to the Pittsburgh Healthy Heart Project, evaluating the somatic–vegetative symptoms of depression using the Beck Depression Inventory-II can predict increases in IR over time in middle-aged to elderly respondents [15].

IR occurs as part of a cluster of cardiovascular–metabolic abnormalities. Given the seriousness of the cardiovascular disease (CVD) data, there is an increasing burden of diabetes. In 2010, its global prevalence was estimated to be 6.4%. The incidence of diabetes among adults will rise between 2010 and 2030, as indicated by the 73% increase in the number of adults with diabetes in developing countries, compared to the 20% increase in developed countries [16]. In this light, IR is a crucial mechanism. It can be defined as a low biological response to normal insulin concentrations. According to this definition, it may relate to many biological actions of insulin in different tissues of the body. In clinical practice, IR usually refers to a state in which a given concentration of insulin is associated with a poor glucose response [17]. The primary cause of metabolic syndrome is IR [3], which in turn reflects a real variety of risk factors, closely linked to each other. Depending on the genetic history of the person developing IR, this cluster of anomalies may contribute to the accelerated development of atherosclerosis, arterial hypertension, or polycystic ovarian syndrome [18,19].

Therefore, we asked the following research questions: (1) Are greater concentrations of thyroid hormones associated with IR in the total sample; (2) is a higher level of depression symptoms associated with IR in the total sample; and (3) do these associations differ between HOMA-IR? We tested these hypotheses using cross-sectional data from a randomized epidemiological study.

The aim of the study was to investigate insulin resistance in association with thyroid function, psychoemotional state, and cardiovascular risk factors among 45–84-year-old citizens of Palanga.

## 2. Materials and Methods

### 2.1. Study Participants

Data were available from a randomized epidemiological study performed in 2013. An independent random sample of 850 citizens of Palanga aged 45–84 years was drawn from the National Population Register. The potential respondents were contacted by phone. The research was accepted by the Lithuanian Bioethics Committee (protocol code: No. BE-2-25). Informed consent was obtained from all participants before the survey. Citizens of Palanga were selected as an object of investigation because they represent a close community with minor migration reflecting the population of the western part of Lithuania. There are no epidemiological data on IR, thyroid function, psychoemotional state, or cardiovascular risk factors in this region of Lithuania.

### 2.2. Study Procedure

All study participants were evaluated according to socio-demographic characteristics (i.e., age, gender, height, weight, waist circumference, education, marital status, and type of job), behavioral factors, and self-perceived health using a questionnaire. Fasting blood samples were drawn from all participants, and biochemical tests were performed for the glucose, insulin, and thyroid hormone concentrations; total cholesterol; low-density lipoprotein (LDL); high-density lipoprotein (HDL); and triglyceride. The psychoemotional state was assessed using the Hospital Anxiety and Depression Scale (HADS) [20] and WHO well-being test [21], while CVD and cardiovascular condition were assessed by a cardiologist using a convenient examination. CVD form was determined via electrocardiogram (ECG) examination and testing using Rose’s questionnaire [22]. ECG data were evaluated using the Minnesota encoding and interpreting system. Metabolic syndrome (MetS) was defined according to the Adult Treatment Panel III (ATPIII) [23] and International Diabetes Federation (IDF) [24] criteria. Under the ATPIII criteria, MetS was defined as the presence of three or more of the following risk factors: abdominal obesity (waist circumference ≥102 cm (men) or ≥88 cm (women)), triglyceride ≥1.7 mmol/L (150 mg/dL), HDL-cholesterol <1.03 mmol/L (40 mg/dL) in men or <1.30 mmol/L (50 mg/dL) in women, fasting glucose ≥6.1 mmol/L (110 mg/dL), and systolic blood pressure >130 mm Hg or diastolic blood pressure >85 mm Hg. MetS was identified when three or more of the five components mentioned above were present.

Further inquiries regarding original questionnaires employed in the study can be directed to the corresponding author.

### 2.3. Measures

#### 2.3.1. WHO-5 Well-Being Test

The WHO-5 Well-being Index [21] questionnaire contains five questions reflecting the well-being of a person during the last 2 weeks: I feel cheerful and in good spirits; I feel calm and relaxed; I feel active and vigorous; I wake up feeling fresh and rested; my daily life is filled with things that interest me. The raw score is calculated by totaling the figures of the five answers. The raw score ranges from 0 to 25, 0 representing worst possible and 25 representing best possible quality of life. To obtain a standardized percentage score ranging from 0 to 100, the raw score is multiplied by 4. A standardized score of 0 represents worst possible whereas a score of 100 represents best possible quality of life. Cronbach α = 0.876. Respondents who scored 50 or more were considered to not have depressive mood. For the respondents who score less than 50, depressive mood was identified, and they were ascribed to the group with increased risk of depression.

#### 2.3.2. Hospital Anxiety and Depression Scale

The HADS is a self-reported questionnaire with 14 items assessing symptoms of general anxiety (HADS-A) and depression (HADS-D) [20]. Total scores on the HADS-A and HADS-D range from 0 to 21, with higher scores suggesting stronger general anxiety or depression. Good internal consistency of HADS was observed in our study sample. For the HADS-D, Cronbach α = 0.752; for the HADS-A, Cronbach α = 0.854.

#### 2.3.3. A Questionnaire on General Data, Behavioral Factors, and Self-Perceived Health

A questionnaire on general data [25] was used to collect information about the marital status, education, employment, and income of respondents. The questionnaire on behavioral factors [25] contained questions about smoking, alcohol consumption, and physical activity during the last year. The questionnaire on self-perceived health [25] contained questions about complaints and diagnosed diseases, medicines used during the last year, frequency of stress events, and visits to any doctor.

#### 2.3.4. Objective Investigation

Arterial blood pressure (mmHg) was measured twice using a quicksilver sphygmomanometer on the right hand while a person was sitting, with the precision of 2 mm referring to the methodological recommendations [26]. The average of the two measurements was used for the analysis. The participants were classified as hypertensive if their systolic blood pressure was ≥140 mmHg and/or diastolic arterial blood pressure was ≥90 mmHg, or if they received antihypertensive drug treatment in the last two weeks.

#### 2.3.5. Weight, Height, and Waist Circumference Measurement

Body height was measured in stockinged feet (without shoes) using a medical height rod. Body weight was measured without shoes using a medical scale. Body mass index (BMI) was calculated according to the formula BMI = body mass (kg)/height^2^ (m) using the data from height and weight measurements. Overweight was diagnosed when BMI was 25.0–29.9 kg/m^2^, and obesity was diagnosed when BMI was 30.0 kg/m^2^ or more. Waist circumference was measured during the physician examination. Before the weight and height were measured, with participants standing without shoes or heavy outer garments, waist circumference was measured in the erect position at the midpoint between the lowest rib and the superior border of the iliac crest.

#### 2.3.6. Rose’s Questionnaire

The standard questionnaire developed by Rose for the measurement of chest pain on exertion consists of eight test-type questions with possible answers [22]. The Rose chest pain questionnaire is recommended as a measure of the prevalence of ischemic cardiac symptoms. The questionnaire measures symptoms, rather than the presence of disease, and their validity as measures of disease in the population under study. Based on the answers, the patients were classified as (1) without angina; (2) having definite angina; and (3) having possible angina. Patients were only considered to have angina if they met all “definite angina” criteria. In addition, all participants underwent an electrocardiogram.

#### 2.3.7. Laboratory Tests

Blood serum analyses were performed in a biochemical testing laboratory. The participants had been warned in advance to come having fasted for at least 12 h. Blood was taken from the elbow vein directly into vacuum blood collection systems, while the person was sitting (amount: 7 mL). The serum was separated from the blood by centrifugation at 3000× *g* and then frozen at –70 °C. The serum levels of the biochemical samples were analyzed via electrochemiluminescence immunoassay (Advia Centaur XP 2016; Siemens Osakeyhtio, Espoo, Finland). Blood samples were analyzed for concentrations of glucose (mmol/L), norm (4.1–5.9); insulin (mU/L), norm (3.0–25.0); TSH (mIU/L), norm (0.55–4.78); FT4 (pmol/L), norm (11.5–22.7); FT3 (pmol/L), norm (3.5–6.5); thyroid peroxidase antibodies (Anti-TPO, U/mL), norm (<60); total cholesterol (mmol/L), norm (2.6–5.2); LDL cholesterol (mmol/L), norm (2.6–3.4); HDL cholesterol (mmol/L), norm in men (0.9–1.7), norm in women (0.9–2.0); and triglyceride (mmol/L), norm (0.5–2.3). IR was calculated according to the HOMA-IR formula (HOMA-IR = (fasting plasma insulin [μIU/mL]) × (fasting plasma glucose [mmol/L])/22.5); the normal rate of HOMA is ≤2.7.

### 2.4. Statistical Analysis

The clinical and sociodemographic characteristics are reported as frequencies and percentages for the categorical variables, with means and standard deviations for the continuous variables, and as medians (25th–75th percentiles) for variables with non-normal distribution. The similarity of the variable distribution to normal was assessed visually and using the Kolmogorov–Smirnov and Shapiro–Wilk tests. The data characteristics were compared between groups without HOMA-IR and with HOMA-IR using Fisher’s χ^2^ test, the parametric two-tailed Student’s *t*-test, or nonparametric Mann–Whitney U test. The thyroid parameters (TSH, FT4, FT3, and FT3/FT4) were divided into quartiles (Q1–Q4 (highest)) with the presence of MetS. The sensitivity, specificity, and predictive values were calculated for the HOMA-IR scores to define the best cut-off point when screening for possible T2DM. The area under the receiver performance characteristic (ROC) curve (AUC) was used as a summary measure of the capacity of the HOMA-IR to detect T2DM. Statistical analyses were performed using the Statistical Package for the Social Sciences software v.22 (SPSS, Chicago, IL, USA). The level of significance was set at *p* < 0.05.

## 3. Results

### 3.1. Baseline Characteristics

Table 1 lists the socio-demographic, clinical, psychoemotional, cardiovascular risk factor, and thyroid biomarker characteristics of all 835 participants stratified into groups without IR (HOMA-IR ≤ 2.7; 67%, *n* = 557) and with IR (HOMA-IR > 2.7; 33%, *n* = 278). Approximately 1.8% (*n* = 15) of the participants were excluded from the analysis due to a history of thyroid disease. In short, the mean age of the study population was 63.6 ± 10.3 years. The majority of participants (64%, *n* = 535) were female, and 67% (*n* = 557) were married. Of the participants, 68% (*n* = 565) had not obtained higher education.

As demonstrated in Table 1, significant differences in age, gender, and education were found between the different study groups. Participants with IR were more likely to be older, be male, and have not obtained higher education.

### 3.2. Psychoemotional State

The proportion of participants with bad well-being was 33%, as determined using the WHO well-being test; anxiety symptoms were shown by 33% of participants, and depression symptoms were shown by 20%, as measured by HADS. However, there were no significant differences in psychoemotional state between the study groups (Table 2).

### 3.3. Cardiovascular Risk Factors

As presented in Table 3, the analysis of parameters between the two study groups distributed according to HOMA-IR showed some statistically significant relationships between IR and cardiovascular risk factors. Individuals with IR were characterized by significantly higher arterial blood pressure, both systolic and diastolic, and by more frequently diagnosed arterial hypertension. A significantly higher BMI was characteristic for the IR group; in addition, waist circumference, both in men and in women, was significantly higher in the IR group. Furthermore, participants with IR were more likely to have lower physical activity measured by the self-evaluated questionnaire, along with higher mean fasting glucose and mean fasting insulin. The IR group also had significantly higher incidence rates of MetS and diagnosed T2DM. There was an observed relationship among diagnosed cardiovascular diseases and IR. AH and coronary artery disease (CAD) together with AH were more frequently observed in those with IR. Conversely, CAD as a separate disease without AH was more frequently observed in the group without IR. This might be explained by insufficient numbers of cases in both groups.

### 3.4. Thyroid Biomarkers

No significant differences were found in the assessment of FT4, FT3, TSH, and anti-TPO between study groups in respect to IR (Table 4).

### 3.5. Subject Groups According to Symptoms of Metabolic Syndrome

When the subjects were divided into groups according to symptoms of MetS, it was found that the TSH and FT3 concentrations and the FT3/FT4 ratio differed significantly between the groups. Significantly higher TSH and lower FT3 were found in the group with MetS. Not all patients in the group with MetS had IR (HOMA-IR > 2.7). Only 70.1% of subjects in the MetS group and 21.4% in the non-MetS group had IR (Table 5).

### 3.6. Thyroid Parameters Divided into Quartiles

We correlated the thyroid parameters (divided into quartiles) with the presence of MetS. Subjects in the lowest (first) quartile for FT3 (<4.8 pmol/L) and FT3/FT4 ratio (<0.31) were significantly more likely to have MetS (32.6% and 31.4%, respectively) compared with those in higher quartiles (Table 6). There was no significant correlation of MetS with TSH and FT4 in the groups divided into quartiles.

### 3.7. HOMA-IR Cut-Offs for Diagnosing T2DM

The HOMA-IR cut-offs for diagnosing T2DM are shown in Table 7. When the HOMA-IR score is higher than 3.45, all subjects are significantly more likely to have T2DM. In men, the cut-off was higher (3.52), with higher sensitivity (94.1) but slightly lower specificity (79.9) compared with those for women (respectively, 3.35, 65.0, and 84.4). These data demonstrated an invariant relationship between HOMA-IR and the presence of T2DM.

**Figure 1:**
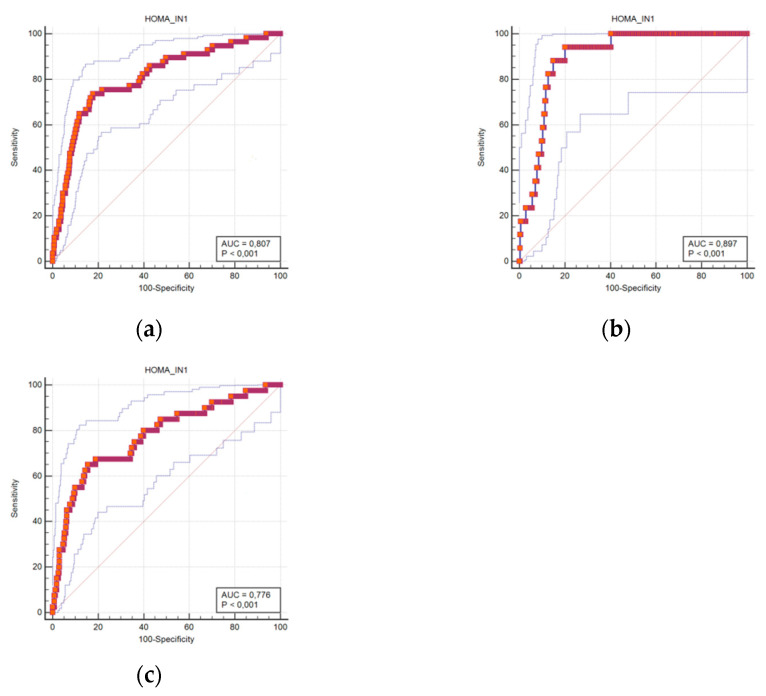
Receiver operating characteristic curves for DM prediction using the HOMA-IR score cut-offs in all subjects (**a**), men (**b**), and women (**c**).

The predictable accuracy is presented using ROC curves. The ROC curves for HOMA-IR scores in all subjects, and in women and men separately, are shown in Figure 1A–C. The curves suggest that the predictive ability of HOMA-IR in diagnosing T2DM is significant.

The areas under the ROC curve using the HOMA-IR cut-off for diagnosing T2DM were 0.807 in the all-subjects group (95% confidence interval (CI) 0.779–0.834), 0.897 in men (95% CI 0.857–0.929), and 0.776 in women (95% CI 0.738–0.810).

## 4. Discussion

If individuals with IR have a high risk of diabetes or MetS, they are more likely to have depression even in normal and prediabetes conditions [1,2,3,15]. Furthermore, IR by itself is a major cardiovascular risk factor in healthy persons. Therefore, in this study, we aimed to investigate IR in association with thyroid function, psychoemotional state, and cardiovascular risk factors among 45–84-year-old citizens of Palanga, which is located in the western part of Lithuania. The present study has three major findings: First, IR showed no association with depressive symptoms as assessed by the HADS and WHO well-being test; second, the subgroup analysis revealed that IR was more likely to occur in male participants, those above the age of 60, and those without higher education; third, IR was strongly associated with the presence of cardiovascular risk factors but not with thyroid biomarkers in the population.

In terms of IR assessment, most of the foregoing studies assessed IR using HOMA-IR; however, various methods were used to assess depressive symptoms across the studies. In the present study, we used the HADS because of its well-established validity and reliability in evaluating depressive symptoms in various populations [27,28]. Our results are not concordant with those of previous studies reporting a link between IR and depression. In contrast to our study, a study conducted by Adriaanse et al. (2006) [14] demonstrated that depressive symptoms were weakly associated with IR in a study sample of 541 participants aged 55–75 years. A study by Lawlor et al. (2005) [29] found that IR was not associated with reduced depressive symptoms in a prospective study of middle-aged men. This contradicted their earlier findings in a cross-sectional study of older women, in which they found a positive association between insulin resistance and depression as assessed using Beck’s depression inventory [30].

The relative importance of IR remains undefined as to the stratification of cardiovascular risk in the population, and the interactions between hyperglycemia, IR, and cardiovascular risk factors are complex and still underestimated [31]. In our study, traditional cardiovascular risk factors such as obesity, evaluated by waist circumference and BMI, low physical activity, and arterial blood pressure were observed statistically significantly more often in subjects with IR. These factors might be responsible for statistically significantly higher numbers of cases of MetS and diagnosed T2DM, as well as for the higher incidence of CVD (AH and CAD), in the IR group. Our findings are supported by a study of 902 nondiabetic subjects between 30 and 80 years of age, recruited from a cross-sectional population-based study in Telde, Gran Canaria Island, Spain, which also demonstrated that individuals with impaired glucose tolerance had increased values of cardiovascular risk factors and higher indexes of IR [32]. Other experimental and clinical studies clearly demonstrated that glucose levels and impaired insulin signaling are potent drivers of the atherosclerotic process, even in the absence of concomitant risk factors such as hypertension, obesity, and dyslipidemia [33]. We found a dramatically increased number of subjects with a fasting glucose level of ≥6.1 mmol/L in the IR group, which is indicative of IR and cardiovascular risk factors [32]. The total cholesterol level as a cardiovascular risk factor did not differ between groups with and without IR; however, the level of triglycerides, which is the predictor for CAD, was statistically significantly higher in subjects with IR. The impact of IR as an individual cardiovascular risk factor in diabetic patients has emerged only recently [34,35]. IR is not only a consequence of obesity without any active role in the etiology of diabetic cardiovascular complications. Several experimental studies have shown that the loss of insulin signaling in the endothelium leads to vascular dysfunction, the expression of adhesion molecules, and atherosclerotic lesions in mice [36,37]. IR is not only an epiphenomenon of obesity. IR is as a key player in the pathophysiology of MetS, which significantly increases cardiovascular risk among patients with T2DM [38]. Our data suggest that an increase in fasting glucose and higher incidence rates of MetS, diabetes, and CVD in subjects with IR are associated with greater prevalence of cardiovascular risk factors.

It is important to note that previous studies in the literature have detected several issues regarding the significant positive correlation between HOMA-IR and blood TSH levels [39,40,41,42,43] and positive correlations with FT4 [42,44,45] and FT3 [42,45,46,47]. Other studies reported completely opposite results, such as a negative correlation between TSH [44], FT4 [41], FT3 [48], and HOMA-IR. Most of these studies found only one hormone association with HOMA-IR, some found a pair of hormone associations, and several found no correlation [49,50]. Based on our study results, there was no significant association between TSH, FT4, FT3, and HOMA-IR. Also, there was no association between increased anti-TPO and HOMA-IR. Therefore, the relationship between thyroid hormones and IR remains unclear. However, it could not be excluded that thyroid function may influence insulin sensitivity. Meanwhile, IR plays a key role in MetS. There are many published data in the scientific literature showing a strong relationship between MetS and TSH and FT3 levels. Some of our results also showed an association between MetS and thyroid hormones. Significantly lower FT3 and FT3/FT4 ratio and higher TSH were found in the group with MetS. We did not find any differences in FT4 levels between the groups. Some similar results were found in other studies. Many authors reported an association of MetS with increased TSH [40,43,47,49,50,51,52,53,54,55], but a relationship between FT4 and MetS was not always obtained, or contradictory results were found [39,52,56,57,58,59]. Some authors have reported a relationship between MetS and higher FT3; this is in contrast to our results [46,47,48,57]. This is consistent with many studies that have demonstrated an association of obesity with thyroid function and especially with higher TSH level. In our study, subjects with the lowest FT3 concentration (Q1) and FT3/FT4 ratio (Q1) were more likely to have MetS. However, Wolffenbuttel, B.H.R., et al. published the exact opposite results to our findings [60]. These data are consistent with the results of many studies that have shown an association between obesity and thyroid function, whereas obesity is directly related to MetS [6,8,9,50,52].

Furthermore, the findings indicate the presence of a HOMA-IR cut-off signaling established IR. This condition is one of the main pathogenetic mechanisms of T2DM among wealthy populations [1]. The evaluation of IR through HOMA-IR is a key index for the primary prevention of T2DM and is thus found in guidelines for the screening of high-risk groups [61]. Knowing HOMA-IR cut-offs is essential for differentiating healthy individuals from those with IR. Therefore, the present study aimed to determine the HOMA-IR cut-off for Lithuania. Although IR is usually defined as a value greater than the 75th percentile value for non-diabetic subjects, the cut-off values reported in the literature vary widely [61]. The results from the present study are very similar to those reported in a study defining HOMA-IR cut-offs for middle-aged men and women in the Czech Republic [62]. In a cross-sectional study of Chinese people, Lee et al. [63] derived an optimal HOMA-IR cut-off of 2.0 to discriminate nondiabetics from diabetics. In our study, in men, the cut-off was higher (3.52), with higher sensitivity (94.1) but slightly lower specificity (79.9) compared with those for women (respectively, 3.35, 65.0, and 84.4).

In addition, it is important to note that the results of this study suggest the existence of HOMA-IR cut-offs that can predict the presence of T2DM, and HOMA-IR can be used not only to indicate the presence of IR. The introduction of this marker into clinical practice may help to prevent T2DM.

## 5. Conclusions

In conclusion, an increase in fasting glucose and more frequent incidence of metabolic syndrome, diabetes, and cardiovascular diseases in subjects with IR are associated with the prevalence of cardiovascular risk factors.

No significant association was found between thyroid function and HOMA-IR.

Our data show the existence of HOMA-IR cut-offs that can predict the presence of T2DM, so HOMA-IR can be used not only to indicate the presence of IR. The introduction of this marker into clinical practice may help to prevent T2DM.

## Figures and Tables

**Table 1:** The sociodemographic characteristics of subject groups without and with insulin resistance (IR).

Baseline Characteristics	Total (*n* = 835)	Without-IR Group	With-IR Group	*p*-Value
		HOMA-IR ≤ 2.7, (*n* = 557)	HOMA-IR > 2.7, (*n* = 278)	
Age, years; mean ± SD	63.6 ± 10.3	63.0 ± 10.5	64.8 ± 9.7	**0.015**
Gender; *n* (%)				**0.003**
Male	300 (35.9)	181 (32.5)	119 (42.8)	
Female	535 (64.1)	376 (67.5)	159 (57.2)	
Marital status; *n* (%)				0.533
Married	557 (66.7)	376 (67.5)	181 (65.1)	
Alone	278 (33.3)	181 (32.5)	97 (34.9)	
Education; *n* (%)				**0.001**
Lower than higher	565 (67.7)	351 (63.0)	214 (77.0)	
Higher	270 (32.3)	206 (37.0)	64 (23.0)	
Employment; *n* (%)				0.142
Employed	400 (47.9)	277 (49.7)	123 (44.2)	
No employed	435 (52.1)	280 (50.3)	155 (55.8)	

Note: HOMA-IR—homeostatic model assessment of insulin resistance. *p* value of probability for comparison between groups (bolded numbers indicate significant differences, *p* < 0.05); data presented as *n* (%), mean ± SD.

**Table 2:** The psychoemotional state characteristics of subject groups without and with insulin resistance (IR).

Psychoemotional State Characteristics	Total (*n* = 835)	Without-IR Group	With-IR Group	*p*-Value
		HOMA-IR ≤ 2.7, (*n* = 557)	HOMA-IR > 2.7, (*n* = 278)	
WHO-5 scores; mean ± SD	55.9 ± 18.2	55.8 ± 18.3	55.9 ± 18.2	0.942
WHO-5 with bad possible quality of life; *n* (%)	276 (33.1)	186 (33.4)	90 (32.4)	0.815
HADS-A scores; median (IQR)	6.0 (3.0–9.0)	6.0 (3.0–9.0)	6.0 (3.0–9.0)	0.810
HADS-D scores, median (IQR)	4.0 (2.0–7.0)	4.0 (2.0–7.0)	4.5 (2.0–7.0)	0.291
HADS-A ≥ 8; *n* (%)	300 (36.1)	202 (36.4)	98 (35.4)	0.818
HADS-D ≥ 8; *n* (%)	170 (20.4)	104 (18.7)	66 (23.8)	0.100
HADS with anxiety and depression ≥ 8; *n* (%)	124 (14.9)	81 (14.5)	43 (15.5)	0.757

Note: HOMA-IR—homeostatic model assessment of insulin resistance; WHO-5—well-being index; HADS-A—Anxiety subscale of Hospital Anxiety and Depression Scale; HADS-D—Depression subscale of Hospital Anxiety and Depression Scale. *p* value of probability for comparison between groups (significance level was *p <* 0.05); data presented as *n* (%), mean ± SD and median (IQR)—25–75 percentiles.

**Table 3:** The cardiovascular risk factor characteristics of subject groups without and with IR.

Cardiovascular Risk Factors Characteristics	Total (*n* = 835)	Without-IR Group	With-IR GROUP	*p*-Value
		HOMA-IR ≤ 2.7, (*n* = 557)	HOMA-IR > 2.7, (*n* = 278)	
Systolic blood pressure, mm Hg; mean ± SD	129.5 ± 12.9	128.3 ± 12.9	132.2 ± 12.5	**<0.001**
Diastolic blood pressure, mm Hg; mean ± SD	74.6 ± 7.3	74.0 ± 7.2	75.7 ± 7.4	**0.002**
Presence of Disease; *n* (%)				**0.001**
Without AH and CAD	159 (19.0)	122 (21.9)	37 (13.3)	
AH	398 (47.7)	256 (46.0)	142 (51.1)	
CAD	43 (5.1)	35 (6.3)	8 (2.9)	
With AH and CAD	235 (28.1)	144 (25.9)	91 (32.9)	
Rose questionnaire: angina pectoris; *n* (%)	88 (10.5)	51 (9.2)	37 (13.3)	0.065
BMI, kg/m^2^; mean ± SD	28.1 ± 4.8	26.6 ± 4.1	31.0 ± 4.7	**<0.001**
BMI ≥30; *n* (%)	261 (31.3)	99 (17.8)	162 (58.3)	**<0.001**
Waist circumference men’s, cm; mean ± SD	98.8 ± 11.2	93.9 ± 9.8	104.8 ± 9.8	**<0.001**
Waist circumference women’s, cm; mean ± SD	88.7 ± 12.7	84.6 ± 10.6	98.5 ± 11.7	**<0.001**
Low physical activity; *n* (%)	239 (29.7)	145 (26.9)	94 (35.2)	**0.015**
Total cholesterol, mmol/L; mean ± SD	6.0 ± 1.2	6.0 ± 1.1	6.0 ± 1.2	0.778
LDL, mmol/L; mean ± SD	3.8 ± 1.1	3.8 ± 1.0	3.9 ± 1.1	0.356
HDL men’s, mmol/L; mean ± SD	1.5 ± 0.43	1.6 ± 0.43	1.3 ± 0.37	**<0.001**
HDL women’s, mmol/L; mean ± SD	1.78 ± 0.48	1.9 ± 0.46	1.6 ± 0.47	**<0.001**
Triglyceride, mmol/L; mean ± SD	1.4 ± 0.70	1.2 ± 0.58	1.7 ± 0.83	**<0.001**
With metabolic syndrome; *n* (%)	204 (24.5)	61 (11.0)	143 (51.4)	**<0.001**
Smoking regular; *n* (%)	123 (14.7)	85 (15,3)	38 (13.7)	0.541
Type 2 diabetes mellitus; *n* (%)	62 (7.4)	17 (3.1)	45 (16.2)	**<0.001**
Fasting glucose, mmol/L; mean ± SD	5.4 ± 1.1	5.1 ± 0.47	6.1 ± 1.7	**<0.001**
Fasting glucose ≥ 6.1; *n* (%)	95 (11.4)	8 (1.4)	87 (31.3)	**<0.001**
Fasting insulin, mU/L; mean ± SD	5.4 ± 1.1	7.4 ± 2.4	16.9 ± 6.8	**<0.001**

Note: HOMA-IR—homeostatic model assessment of insulin resistance; BMI—body mass index; AH—arterial hypertension; CAD—coronary artery disease; LDL—*low*-density lipoprotein cholesterol; HDL—high-density lipoprotein cholesterol. *p* value of probability for comparison between groups (bolded numbers indicate significant differences, *p* < 0.05); data presented as *n* (%), mean ± SD.

**Table 4:** The thyroid biomarker characteristics of subject groups without and with IR.

Thyroid Biomarkers Characteristics	Total (*n* = 835)	Without-IR Group	With-IR Group	*p*-Value
		HOMA-IR ≤ 2.7, (*n* = 557)	HOMA-IR > 2.7, (*n* = 278)	
TSH, mIU/L; median (IQR)	2.0 (1.4–3.1)	2.1 (1.4–3.2)	2.0 (1.3–3.0)	0.338
TSH; *n* (%)				0.494
<0.55	25 (3.0)	14 (2.5)	11 (4.0)	
0.55–4.78	740 (88.6)	495 (88.9)	245 (88.1)	
>4.78	70 (8.4)	48 (8.6)	22 (7.9)	
Anti-TPO, U/mL; median (IQR)	51.8 (44.2–62.7)	50.9 (43.6–63.7)	53.5 (45.5–62.3)	0.316
Anti-TPO; *n* (%)				0.676
0–60	596 (71.4)	395 (70.9)	201 (72.3)	
>60	239 (28.6)	162 (29.1)	77 (27.7)	
FT3, pmol/L; (mean ± SD)	5.2 ± 0.66	5.2 ± 0.62	5.2 ± 0.74	0.431
FT3; *n* (%)				0.870
≤6.5	815 (97.6)	544 (97.7)	271 (97.5)	
>6.5	20 (2.5)	13 (2.3)	7 (2.5)	
FT4, pmol/L; (mean ± SD)	15.5 ± 2.1	15.4 ± 2.1	15.6 ± 2.0	0.348
FT4, *n* (%)				0.870
11.5–22.7	835 (100)	557 (100)	278 (100)	

Note: Note: HOMA-IR—homeostatic model assessment of insulin resistance; TSH—thyroid stimulating hormone; Anti-TPO—thyroid peroxides antibodies; FT3—free triiodothyronine; FT4—free tetraiodothyronine. *p* value of probability for comparison between groups (significance level was *p <* 0.05); data presented as *n* (%), mean ± SD and median (IQR)—25–75 percentiles.

**Table 5:** The biochemical and homeostasis model assessment of IR (HOMA-IR) parameters of subject groups without and with metabolic syndrome.

Parameter	All	Without MetS, *n* = 631	With MetS, *n* = 204	*p*
TSH, mIU/L; median (IQR)	2.0 (1.4–3.1)	2.0 (1.3–3.0)	2.3 (1.5–3.3)	**0.049**
TSH; *n* (%)				0.339
<0.55	25 (3.0)	21 (3.3)	4 (2.0)	
0.55–4.78	740 (88.6)	561 (88.9)	179 (87.7)	
>4.78	70 (8.4)	49 (7.8)	21 (10.3)	
Anti-TPO, U/mL; median (IQR)	51.8 (44.2–62.7)	51.3 (43.8–62.2)	53.1 (45.4–63.9)	0.235
Anti-TPO; *n* (%)				0.423
0–60	596 (71.4)	455 (72.1)	141 (69.1)	
>60	239 (28.6)	176 (27.9)	63 (30.9)	
FT3, pmol/L; (mean ± SD)	5.2 ± 0.66	5.2 ± 0.65	5.1 ± 0.70	**0.025**
FT3; *n* (%)				0.870
≤6.5	815 (97.6)	616 (97.6)	199 (97.5)	
>6.5	20 (2.5)	15 (2.4)	5 (2.5)	
FT4, pmol/L; (mean ± SD)	15.5 ± 2.1	15.5 ± 2.1	15.6 ± 2.0	0.574
FT4, *n* (%)				0.870
11.5–22.7	835 (100)	631 (100)	204 (100)	
FT3/FT4	0.34 ± 0.05	0.34 ± 0.05	0.33 ± 0.05	**0.013**
HOMA_IR	2.1 (1.5–3.1)	1.9 (1.3–2.6)	3.7 (2.5–5.1)	**<0.001**
HOMA-IR > 2.7	278 (33.3)	135 (21.4)	143 (70.1)	**<0.001**

Note: HOMA-IR—NotNote: HOMA-IR—homeostatic model assessment of insulin resistance; MetS—metabolic syndrome; TSH—thyroid stimulating hormone; Anti-TPO—thyroid peroxides antibodies; FT3—free triiodothyronine; FT4—free tetraiodothyronine. *p* value of probability for comparison between groups (bolded numbers indicate significant differences, *p* < 0.05); data presented as n (%), mean ± SD and median (IQR)—25–75 percentiles.

**Table 6:** Frequency of metabolic syndrome according to quartiles of thyroid hormone parameters.

Parameter	Q1	Q2	Q3	Q4,	*p*	Q1:Q2	Q1:Q3	Q1:Q4
TSH	*n* = 210	*n* = 208	*n* = 208	*n* = 208				
MetS, *n* (%)	42 (20.0)	51 (24.5)	55 (26.4)	56 (26.9)	0.336	ns	ns	ns
FT3	*n* = 193	*n* = 187	*n* = 211	*n* = 243				
MetS, *n* (%)	63 (32.6)	43 (23.0)	45 (21.3)	53 (21.8)	**0.026**	**0.037**	**0.011**	**0.011**
FT4	*n* = 198	*n* = 205	*n* = 221	*n* = 210				
MetS, *n* (%)	46 (23.2)	52 (25.4)	51 (23.1)	55 (26.2)	0.844	ns	ns	ns
FT3/FT4	*n* = 194	*n* = 214	*n* = 208	*n* = 218				
MetS, *n* (%)	61 (31.4)	56 (26.2)	37 (17.8)	50 (22.9)	**0.013**	ns	**0.002**	0.053

Note: MetS—metabolic syndrome; TSH—thyroid stimulating hormone; Anti-TPO—thyroid peroxides antibodies; FT3—free triiodothyronine; FT4—free tetraiodothyronine; Q1-Q2-Q3-Q4—quartiles of hormones concentrations. Bolded numbers indicate significant differences, *p* < 0.05.

**Table 7:** The homeostasis model assessment of insulin resistance cut-offs for the diagnosis of type 2 diabetes mellitus.

	Cut-Off	Sensitivity	Specificity	Accuracy (95%CI)
HOMA-IR men + women	3.45	73.7	82.3	0.807 (0.779–0.834)
HOMA-IR men	3.52	94.1	79.9	0.897 (0.857–0.929)
HOMA-IR women	3.35	65.0	84.4	0.776 (0.738–0.810)

HOMA-IR—homeostatic model assessment of insulin resistance.
